# Supplementary material for: The mechanosensitive Piezo1 channel mediates heart mechano-chemo transduction
Source: Nat Commun. 2021 Feb 8;12:869. doi: 10.1038/s41467-021-21178-4 (PMC7870949; doi:10.1038/s41467-021-21178-4)
Supplement: Supplementary file 1 — Supplementary Information [file 41467_2021_21178_MOESM1_ESM.pdf]

Supplementary information

The mechanosensitive Piezo1 channel mediates heart mechano-chemo transduction

Fan Jiang[1], Kunlun Yin[2], Kun Wu[1,5], Mingmin Zhang[1], Shiqiang Wang[3], Heping Cheng[4], Zhou Zhou[2], Bailong Xiao[1#]

[1]State Key Laboratory of Membrane Biology, Tsinghua-Peking Center for Life Sciences, Beijing Advanced Innovation Center for Structural Biology, IDG/McGovern Institute for Brain Research, School of Pharmaceutical Sciences, Tsinghua University, Beijing, 100084, China;

[2]State Key Laboratory of Cardiovascular Disease, Beijing Key Laboratory for Molecular Diagnostics of Cardiovascular Diseases, Center of Laboratory Medicine, Fuwai Hospital, National Center for Cardiovascular Diseases, Chinese Academy of Medical Sciences and Peking Union Medical College, Beijing 100037, China;

[3]State Key Lab of Membrane Biology, College of Life Sciences and Institute of Molecular Medicine, Peking University, Beijing, China;

[4]State Key Lab of Membrane Biology, Institute of Molecular Medicine, Peking-Tsinghua Center for Life Sciences, Peking University, Beijing, China;

[5]Current address: Medical Research Center, Beijing Key Laboratory of Cardiopulmonary Cerebral Resuscitation, Department of Emergency, Beijing Chao-Yang Hospital, Capital Medical University, Beijing, China.

[#]To whom correspondence should be addressed:

Bailong Xiao: xbailong@mail.tsinghua.edu.cn, Tel: +86-10-62773981

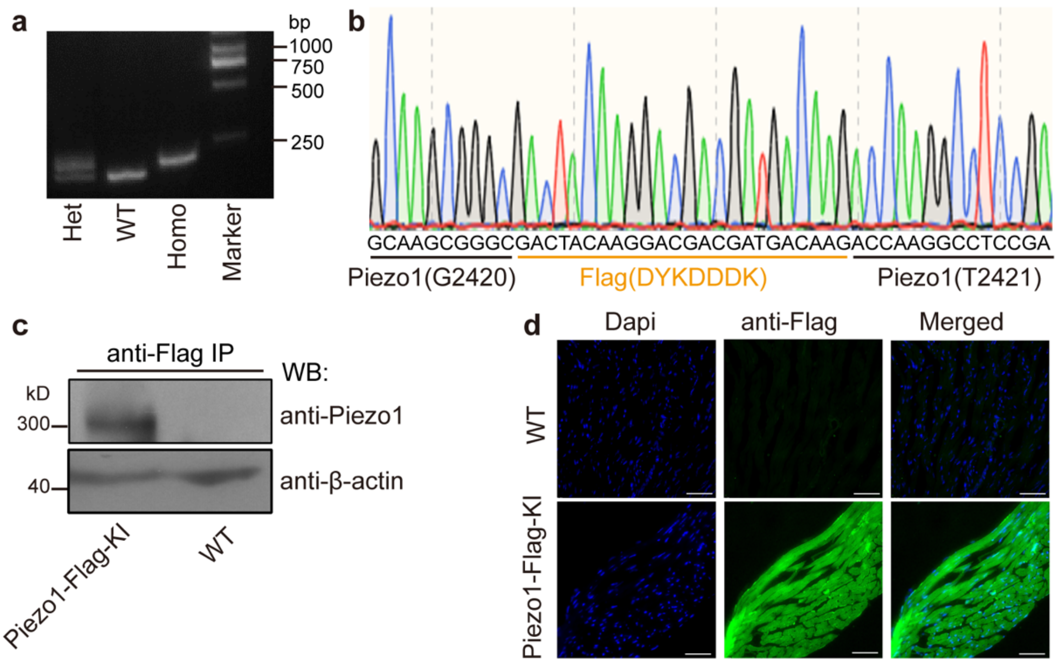

Supplementary Figure 1 Generation and characterization of the Piezo1-Flag-KI mice

a, Genotyping results of the heterozygous (Het) or homozygous (Homo) Piezo1-Flag-KI mice and their wild-type (WT) littermate control mice.

b, DNA sequencing verification of the insertion of the Flag-coding sequence after the genetic sequence coding the residue G2420 of mouse Piezo1.

c, Representative western blotting result of anti-Flag-immunoprecipitated Piezo1-Flag proteins from the Piezo1-Flag-KI and WT littermate control heart homogenates using the anti-Piezo1 antibody. The β-actin level was used for loading control.

d, Representative images showing Dapi staining or immunostaining of heart tissues derived from WT and Piezo1-Flag-KI mice using the anti-Flag antibody. Scale bar, 100μm.

Each experiment was repeated independently three times with similar results.

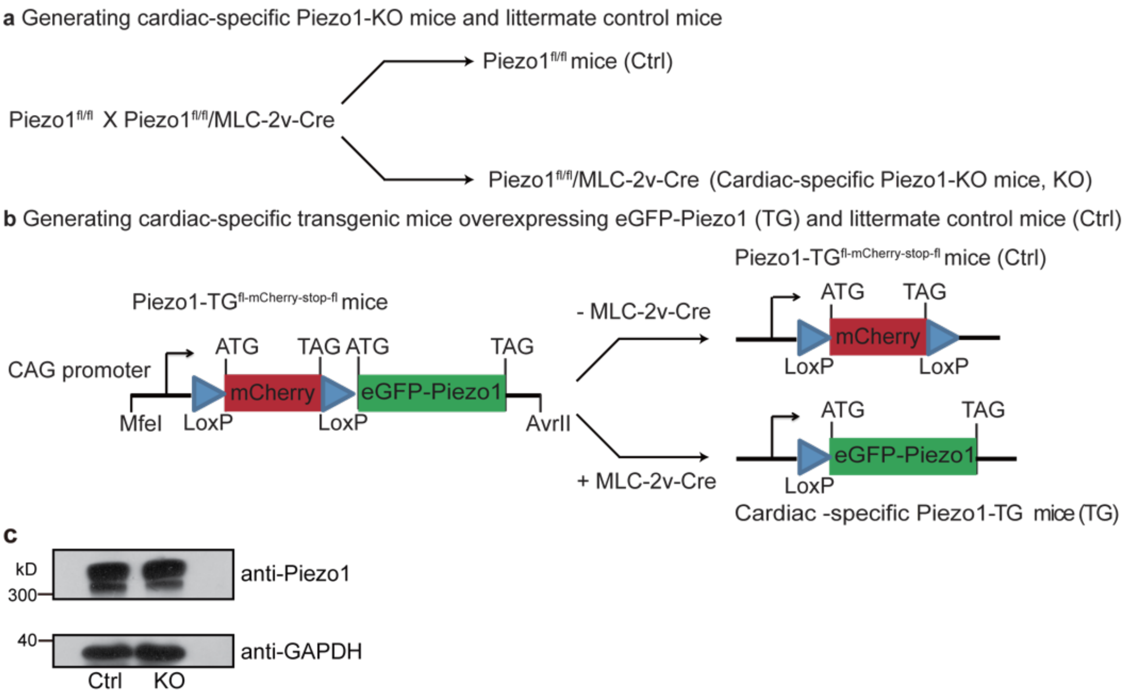

Supplementary Figure 2 Generation of cardiac-specific Piezo1-KO mice and Piezo1-transgenic (Piezo1-TG) mice overexpressing the eGFP-Piezo1 fusion protein

a, Breeding strategy for generating the Piezo1$^{fl/fl}$ littermate control mice and the cardiac-specific Piezo1-KO mice using the MLC-2v-Cre mice.

b, Breeding strategy for generating the Piezo1-TG$^{fl\text{-}mCherry\text{-}stop\text{-}fl}$ littermate control mice (Ctrl) and the cardiac-specific Piezo1-TG mice using the MLC-2v-Cre mice. The Piezo1-TG$^{fl\text{-}mCherry\text{-}stop\text{-}fl}$ mice allow tissue-specific Cre-dependent expression of eGFP-Piezo1. In the absence of Cre recombinase, the upstream floxed mCherry coding sequence with a TAG stop codon blocks the translation of the downstream eGFP-Piezo1 fusion protein, resulting in the Piezo1-TG$^{fl\text{-}mCherry\text{-}stop\text{-}fl}$ littermate control mice that express mCherry. When TG$^{floxed\text{-}mCherry}$ mice are crossed with the cardiac-specific MLC-2v-Cre line, the mCherry sequence is cleaved, leading to generation of the cardiac-specific Piezo1-TG mice that express eGFP-Piezo1 in cardiomyocytes.

c, Representative western blotting result of red blood cells from the KO and Ctrl littermate using the anti-Piezo1 antibody. The GAPDH level was used for loading control. Similar results were obtained from 3 independent experiments.

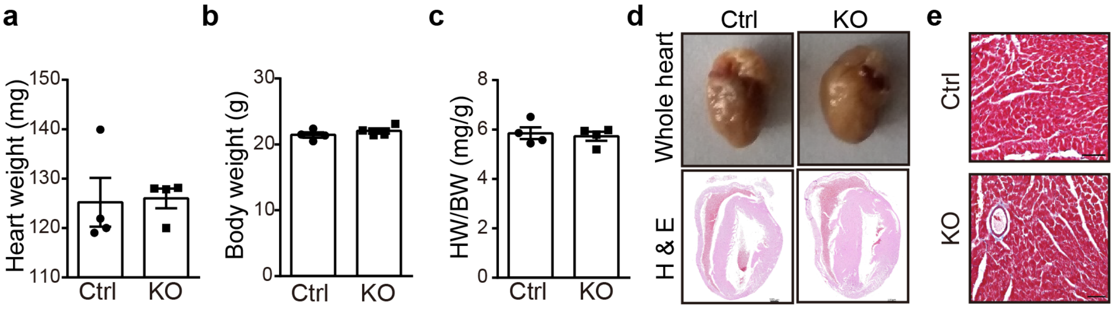

Supplementary Figure 3 8-week-old Piezo1-KO mice show normal heart structure and function

a-**c**, Scatter plot of heart weight (**a**), body weight (**b**), and HW/BW ratio (**c**) of 8-week old (n=4 mice for each group) littermate Ctrl and Piezo1-KO male mice. Unpaired student's t-test. Values are mean ±SEM.

d, Histologic analysis of whole hearts or H & E stained longitudinal heart sections derived from 8-week old littermate Ctrl and Piezo1-KO male mice. Each experiment was repeated independently three times with similar results.

e, Histologic analysis of the left ventricles of 8-week old littermate Ctrl and Piezo1-KO hearts sectioned longitudinally and subjected to Masson's trichrome staining. Each experiment was repeated independently three times with similar results.

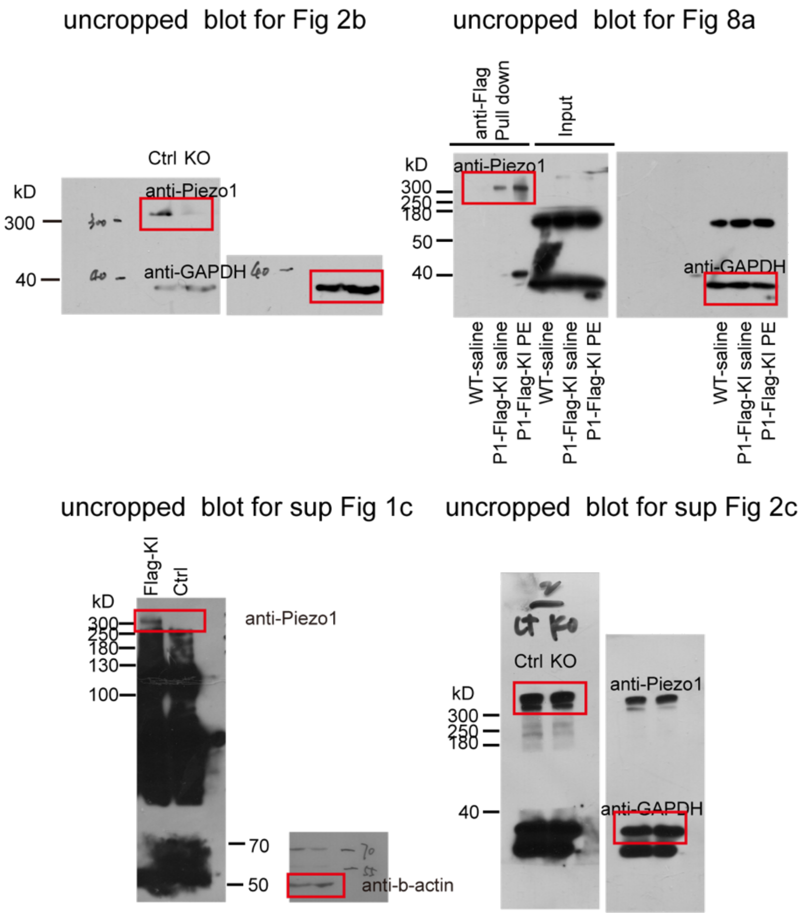

Supplementary Figure 4 Uncropped western blots

Red boxes in the uncropped blots indicate the cropped regions shown in the corresponding figures.

a, Echocardiographic analysis of 8-week old Piezo1-KO and littermate control mice (Ctrl)

	A'(mm/s)	E'(mm/s)	A (mm/s)	E (mm/s)	E'/A'	MV E/A	LVAW;d	LVAW;s	LVID;d	LVID;s	LVPW;d	LVPW;s	EF	FS	LV Mass AW	LV Vol;d	LV Vol;s
Ctrl (n=3)	-18.70 ±2.24	-21.96 ±1.71	432.86 ±50.97	731.45 ±33.98	1.19 ±0.06	1.72 ±0.14	0.63 ±0.05	0.73 ±0.05	3.84 ±0.16	3.06 ±0.19	0.82 ±0.09	0.99 ±0.11	48.60 ±3.02	26.90 ±1.38	96.19 ±8.18	63.66 ±6.09	37.28 ±5.46
KO (n=3)	-20.23 ±3.31	-17.61 ±4.27	478.18 ±95.48	694.98 ±119.49	0.86 ±0.07	1.46 ±0.04	0.71 ±0.01	0.84 ±0.02	3.72 ±0.17	2.68 ±0.08	0.82 ±0.09	1.04 ±0.10	54.83 ±1.63	27.88 ±1.16	97.49 ±0.19	58.98 ±6.45	26.54 ±1.95

Unpaired student's t-test,two-sided. Values are mean ±SEM.

b, Echocardiographic analysis of 18-week old cardio-specific Piezo1-KO and Ctrl mice

	A'(mm/s)	E'(mm/s)	A (mm/s)	E (mm/s)	E'/A'	MV E/A	LVAW;d	LVAW;s	LVID;d	LVID;s	LVPW;d	LVPW;s	EF	FS	LV Mass AW	LV Vol;d	LV Vol;s
Ctrl (n=12)	-17.29±2.11	-24.14±1.95	520.26±26.81	697.80±39.11	1.81±0.37	1.34±0.04	0.89±0.03	1.23±0.08	3.79±0.09	2.48±0.12	0.80±0.04	1.13±0.06	64.27±2.56	34.85±1.93	116.56±5.72	62.14±3.39	22.87±2.55
KO (n=8)	-23.02±1.73	-31.79±1.60*	515.25±40.79	789.66±34.94	1.42±0.09*	1.59±0.14	0.82±0.08	1.17±0.10	4.10±0.24*	3.13±0.20**	0.76±0.02	1.06±0.06	50.94±4.58*	26.21±2.88*	133.42±8.95	81.80±8.70*	40.62±6.27**

Unpaired student's t-test,two-sided. *P < 0.05, **P<0.01. Values are mean ±SEM.

c, Echocardiographic analysis of 4-week old cardio-specific Piezo1-TG and Ctrl mice

	A'(mm/s)	E'(mm/s)	A (mm/s)	E (mm/s)	E'/A'	MV E/A	LVAW;d	LVAW;s	LVID;d	LVID;s	LVPW;d	LVPW;s	EF	FS	LV Mass AW	LV Vol;d	LV Vol;s
Ctrl (n=4)	-20.46 ±5.29	-24.13 ±4.16	238.30 ±36.80	650.99 ±57.48	1.25 ±0.01	2.81 ±0.26	0.83 ±0.04	1.16 ±0.07	3.66 ±0.16	2.43 ±0.22	0.69 ±0.08	0.95 ±0.15	63.17 ±4.45	33.74 ±3.21	93.92 ±5.99	56.90 ±5.92	21.48 ±4.45
TG (n=4)	-20.55 ±2.68	-20.22 ±3.84	395.07 ±91.95	556.70 ±34.36	0.96 ±0.08	1.59 ±0.28*	0.77 ±0.03	0.98 ±0.05	3.76 ±0.03	3.08 ±0.05*	0.70 ±0.06	0.75 ±0.04	37.83 ±3.49*	17.96 ±1.85*	94.34 ±2.68	60.46 ±1.03	37.49 ±1.50*

Unpaired student's t-test,two-sided. *P < 0.05. Values are mean ±SEM.

d, Echocardiographic analysis of 8-week old cardio-specific Piezo1-TG and Ctrl mice

	A'(mm/s)	E'(mm/s)	A (mm/s)	E (mm/s)	E'/A'	MV E/A	LVAW;d	LVAW;s	LVID;d	LVID;s	LVPW;d	LVPW;s	EF	FS	LV Mass AW	LV Vol;d	LV Vol;s
Ctrl (n=4)	-18.41 ±5.84	-22.82 ±5.29	576.50 ±15.31	758.38 ±138.98	1.28 ±0.30	1.10 ±0.16	0.72 ±0.03	1.18 ±0.08	4.11 ±0.21	2.92 ±0.26	0.65 ±0.04	0.91 ±0.07	56.12 ±5.51	29.34 ±3.72	101.00 ±10.37	75.53 ±8.99	34.01 ±6.83
TG (n=4)	-18.95 ±6.21*	-16.91 ±3.53	268.47 ±41.50	665.36 ±109.92	1.05 ±0.18	2.48 ±0.84	0.78 ±0.05	1.09 ±0.10	4.44 ±0.16	3.70 ±0.15*	0.68 ±0.03	0.81 ±0.04	34.52 ±6.80*	16.64 ±3.51*	123.38 ±2.84*	90.27 ±7.57	58.48 ±5.72*

Unpaired student's t-test,two-sided. *P < 0.05. Values are mean ±SEM.

e, Echocardiographic analysis of 18-week old cardio-specific Piezo1-TG and Ctrl mice

	A'(mm/s)	E'(mm/s)	A (mm/s)	E (mm/s)	E'/A'	MV E/A	LVAW;d	LVAW;s	LVID;d	LVID;s	LVPW;d	LVPW;s	EF	FS	LV Mass AW	LV Vol;d	LV Vol;s ul
Ctrl (n=7)	-20.86 ±2.24	-26.29 ±2.37	473.55 ±47.88	751.63 ±40.72	1.25 ±0.12	1.47 ±0.15	0.83 ±0.05	1.32 ±0.04	3.83 ±0.12	2.54 ±0.10	0.75 ±0.04	1.07 ±0.03	63.22 ±2.08	33.82 ±1.48	107.30 ±5.35	63.83 ±4.44	23.56 ±2.20
TG (n= 5)	-16.78 ±1.96	-13.07 ±1.33*	457.75 ±35.40	514.51 ±37.24*	0.63 ±0.04	1.15 ±0.14	0.97 ±0.06	1.29 ±0.05	4.06 ±0.10	3.40 ±0.18**	0.85 ±0.05	0.95 ±0.02*	34.74 ±5.45***	16.57 ±2.81***	144.68 ±13.72*	72.92 ±4.07	48.25 ±6.51**

Unpaired student's t-test,two-sided. *P < 0.05, **P<0.01, ***P<0.001. Values are mean ±SEM.

f, Echocardiographic anaylsis of Dox-induced mouse model

	MV E/A	LVAW;d	LVAW;s	LVID;d	LVID;s	LVPW;d	LVPW;s	EF	FS	LV Mass AW	LV Vol;d	LV Vol;s
Ctrl-Saline (9)	1.42±0.05	0.83±0.03	1.03±0.09	3.80±0.11	2.72±0.17	0.78±0.03	1.02±0.03	60.28±2.94	31.36±2.43	116.67±8.05	68.60±5.99	29.09±4.01
Ctrl-Dox (10)	1.56±0.15	0.84±0.04	1.07±0.08	4.16±0.11*	3.16±0.13	0.76±0.01	0.95±0.03	48.38±2.70**	24.30±1.65*	127.03±9.86	77.54±4.92	40.63±4.17*

Unpaired student's t-test,two-sided. *P < 0.05, **P<0.01. Values are mean ±SEM.

Supplementary Table 1 Echocardiographic analysis of the indicated mice

mANP-qPCR-F	GCCATATTGGAGCAAATCCT
mANP-qPCR-R	GCAGGTTCTTGAAATCCATCA
mβ-MHC-qPCR-F	AAGCAGCAGTTGGATGAGCG
mβ-MHC-qPCR-R	CCTCGATGCGTGCCTGAAGC
mBNP-qPCR-F	CATGGATCTCCTGAAGGTGC
mBNP-qPCR-R	CCTTCAAGAGCTGTCTCTGG
mα-MHC-F	GGAAGAGTGAGCGGCCATCAAGG
mα-MHC-R	CTGCTGGAGAGGTTATTCCTCG
mSERCA2a-F	GAGAACGCTCACACAAAGACC
mSERCA2a-R	CAATTCGTTGGAGCCCCAT
mGAPDH-F	GCACCACCAACTGCTTAG
mGAPDH-R	GGATGCAGGGATGATGTTC
sgRNA-T7-F	taggGTGGGGAGCAAGCGGGCACCA
sgRNA-T7-R	aaacTGGTGCCCGCTTGCTCCCCAC
mP1-Flag-KI-HA-oligo	GTGCGCATCCAGCTGCGGAGGGAGCAAGTGG GCACAGGGGCCTCTGGGGAGCAAGCGGGCGA CTACAAGGACGACGATGACAAGACCAAGGCC TCCGACTTCCTCGAGTGGTGGGTCATCGAGCT GCAGGACTGCAAGGCTGAC
mouse-sequence-F	CCGACTCTAACTATCCCACTCAAC
mouse-sequence-R	CTGACCTTGTCACTGAAGATGACC
Flag-F	GACTACAAGGACGACGATGACAAG
Flag-R	AGGCAGCTCCTTCATTCCCG

Supplementary Table 2 Sequences of primers and sgRNAs
