# Supplementary material for: The mechanosensitive Piezo1 channel mediates heart mechano-chemo transduction
Source: Nat Commun. 2021 Feb 8;12:869. doi: 10.1038/s41467-021-21178-4 (PMC7870949; doi:10.1038/s41467-021-21178-4)
Supplement: Supplementary file 3 — Description of Additional Supplementary Files [file 41467_2021_21178_MOESM3_ESM.docx]

Description of Additional Supplementary Files

File Name: Supplementary Data 1
Description: It showed that the gender, age, disease diagnosis information and examination results. Human heart tissue samples were all from patients with hypothetic cardiomyopathy. All of the patients enrolled in this study were referred by the center of Cardiovascular Surgery in Fuwai hospital. No self-selection bias. The study was approved by the Ethics Committee of Fuwai hospital and adhered to the Declaration of Helsinki. All experimental protocols were approved by the ethics committee of Fuwai hospital, and were carried out in accordance with the approved guidelines.

File Name: Supplementary Movie 1
Description: Representative movies of the long-axis view of left ventricle from 8-week old control mice.

File Name: Supplementary Movie 2
Description: Representative movies of the long-axis view of left ventricle from 8-week old Piezo1-KO mice.

File Name: Supplementary Movie 3
Description: Representative movies of the short-axis view of left ventricle from 8-week old control mice.

File Name: Supplementary Movie 4
Description: Representative movies of the short-axis view of left ventricle from 8-week old Piezo1-KO mice.

File Name: Supplementary Movie 5

Description: Representative movies of the long-axis view of left ventricle from 8-week old control mice.

File Name: Supplementary Movie 6
Description: Representative movies of the long-axis view of left ventricle from 8-week old Piezo1-TG mice.

File Name: Supplementary Movie 7

Description: Representative movies of the short-axis view of left ventricle from 8-week old control mice.

File Name: Supplementary Movie 8
Description: Representative movies of the short-axis view of left ventricle from 8-week old Piezo1-TG mice.
